# Absorption and Metabolism of Phenolics from Digests of Polyphenol-Rich Potato Extracts Using the Caco-2/HepG2 Co-Culture System

**DOI:** 10.3390/foods7010008

**Published:** 2018-01-12

**Authors:** Shima Sadeghi Ekbatan, Michele M. Iskandar, Lekha Sleno, Kebba Sabally, Joelle Khairallah, Satya Prakash, Stan Kubow

**Affiliations:** 1 School of Human Nutrition, McGill University, 21111 Lakeshore, Ste. Anne de Bellevue, Montreal, QC H9X3V9, Canada; shima.sadeghi@mail.mcgill.ca (S.S.E.); michele.iskandar@mail.mcgill.ca (M.M.I.); kebba.sabally@mcgill.ca (K.S.); joelle.khairallah@mail.mcgill.ca (J.K.); 2 Chemistry Department, Université du Québec à Montréal, 2101 Jeanne-Mance, Montreal, QC H2X2J6, Canada; sleno.lekha@uqam.ca; 3 Department of Biomedical Engineering, Duff Medical Building, 3775 Rue University, Montreal, QC H3A2B4, Canada; satya.prakash@mcgill.ca

**Keywords:** in vitro digestion, Caco-2/HepG2 co-culture, potato, chlorogenic acid, ferulic acid, caffeic acid, rutin

## Abstract

The bioactivity of dietary polyphenols depends upon gastrointestinal and hepatic metabolism of secondary microbial phenolic metabolites generated via colonic microbiota-mediated biotransformation. A polyphenol-rich potato extract (PRPE) containing chlorogenic, caffeic, and ferulic acids and rutin was digested in a dynamic multi-reactor gastrointestinal simulator of the human intestinal microbial ecosystem (GI model). Simulated digestion showed extensive degradation of the parent compounds and the generation of microbial phenolic metabolites. To characterize the transport and metabolism of microbial phenolic metabolites following digestion, a co-culture of intestinal Caco-2 and hepatic HepG2 cells was exposed to the PRPE-derived digests obtained from the colonic vessels. Following a 2 h incubation of the digesta with the Caco-2/HepG2 co-cultures, approximately 10–15% of ferulic, dihydrocaffeic, and dihydroferulic acids and 3–5% of 3-hydroxybenzoic, 3-hydroxyphenylpropionic, and coumaric acids were observed in the basolateral side, whereas 3-hydroxyphenylacetic acid, phenylpropanoic acid, and cinnamic acid were not detected. Subsequent HepG2 cellular metabolism led to major increases in ferulic, dihydrocaffeic, 3-hydroxyphenylpropionic, and coumaric acids ranging from 160–370%. These findings highlight the importance of hepatic metabolism towards the generation of secondary metabolites of polyphenols despite low selective Caco-2 cellular uptake of microbial phenolic metabolites.

## 1. Introduction

There is increasing evidence from epidemiological studies and randomized clinical trials showing a strong association between consumption of polyphenols and reduced risk of several chronic diseases such as type 2 diabetes, cardiovascular disease, and some types of cancer [1]. The predominant dietary sources of polyphenols are primarily plant foods that are consumed regularly in large amounts, such as potatoes that are an important food staple in many populations [2]. Potatoes are a good source of common dietary polyphenols—including chlorogenic acid (CGA), caffeic acid (CA), ferulic acid (FA), and rutin (RU)—which are linked with health-promoting properties [3,4]. We have shown that a polyphenol-rich potato extract (PRPE) containing CGA, CA, FA, and RU as the primary polyphenols inhibit the development of glucose intolerance and obesity in the diet-induced obesity mouse model [5]. 

Due to their glycosidic linkages, polyphenols are generally poorly absorbed in the small intestine and so the majority of these compounds reach the colon where glycosides are cleaved by microbiota to generate the aglycon [6]. The aglycons can undergo further hydrolysis, cleavage, and reduction reactions by gut microbiota to produce a variety of small molecular weight end-products that can enter the circulation via transporters or by passive diffusion [7,8,9,10,11]. To study the effects of digestive processes on polyphenol degradation, in vitro digestion models have been used extensively but the majority of those studies have excluded colonic fermentation [1]. To examine the production of colonic microbial metabolites of polyphenols, we utilized a dynamic multi-reactor gastrointestinal simulator of the human intestinal microbial ecosystem (GI) digestion model, which consists of five interconnected vessels representing the stomach and small intestine, as well as reactors pertaining to the ascending, transverse, and descending colon inoculated with human fecal matter [12,13,14]. Our GI model studies have shown that colonic microbiota metabolism of CGA, CA, FA, and RU generates phenylpropionic acids (3-(3,4-dihydroxyphenyl)propionic acid, 3-(phenyl)propionic acid, 3-(4-hydroxy-3-methoxyphenyl)propionic acid), benzoic acids (protocatechuic acid, benzoic acid, vanillic acid, 3-hydroxybenzoic acid), phenylacetic acid (3-hydroxyphenylacetic acid), and cinnamic acids (caffeic acid, dihydrocaffeic acid, ferulic acid, coumaric acid, cinnamic acid) [12]. Identification of microbial phenolic metabolites is pertinent to the bioactivity of polyphenols as several of these secondary microbial phenolic metabolites have demonstrated antioxidant and anti-inflammatory properties via in vitro and in vivo studies [15]. The presence of such metabolites in human plasma has been suggested to be more relevant for the health benefits of polyphenol-rich plant foods than the less bioavailable parent compounds [16]. 

There is limited knowledge regarding cellular uptake of microbial phenolic metabolites due to the complexity of the processes affecting the absorptive processes including diffusion and gut transporters, as well as intestinal and hepatic phase II metabolism [17]. First-pass intestinal and hepatic metabolic reactions involving native polyphenols and microbial phenolic metabolites lead to methylated, glucuronidated, or sulfated derivatives that appear in plasma and urine [11,18]. As processes involved in polyphenol biotransformation and transport have been difficult to fully explore in vivo, recent approaches have coupled in vitro digestion models to double-layered Caco-2 and HepG2 co-cultures [19]. Following treatment of the co-cultures with food digests from GI digestion models, the cell media from the apical and basolateral components were obtained to assess for the biotransformation and transport of metabolites. The human intestinal Caco-2 cell line has been widely used to study the mechanisms of absorption, transport, and metabolism of both drugs and dietary phytochemicals such as polyphenols [20,21]. Caco-2 cells are differentiated when confluence is reached and after 20 days they form monolayers with well-established polarity and tight junctions and express transporter and efflux proteins [22,23]. The human HepG2 cell line is a reliable model that is commonly used to mimic hepatic metabolism of xenobiotics including polyphenols [24]. Combined Caco-2 and liver cell systems have been used for the prediction of oral bioavailability of xenobiotics in animal models [25] and humans [26]. These latter studies have shown very good correlation between the in vitro area under the concentration–time curve and the in vivo bioavailability of the same compound, which indicates that such co-cultures can be used to mimic absorption and first-pass effects. 

The aim of the current study was to use the Caco-2 and HepG2 co-culture system to assess intestinal and hepatic-mediated transport and biotransformation of microbial phenolic metabolites generated from the digestion of PRPE in the GI model. 

## 2. Materials and Methods 

### 2.1. Preparation of the ‘Onaway’ Potato Extract

The potato extract was produced by POS Bio-Sciences (POS Bio-Sciences, Saskatoon, SK, Canada) as previously described [5]. Briefly, ‘Onaway’ potatoes (20 kg) were washed, chopped, and freeze-dried. The extraction was performed by agitating the freeze-dried potatoes with 200 L of a 90% (*v*/*v*) aqueous ethanol solution for 1 h at room temperature. The ratio of powder to aqueous ethanol was 1:10 (*w*/*v*). The extract was then separated from the solids by centrifugation at 1076× *g* for 10 min and concentrated under vacuum at 40–50 °C until a volume of approximately 15 L was reached. During the evaporation, water was added back to lower the ethanol content to less than 10% as measured by a hydrometer. The extract powder was then generated by freeze-drying the concentrate. The phenolic content of the ‘Onaway’ extract in milligram per gram (mg/g) dry matter basis was 8.9 for CGA, 0.6 for CA, 0.2 for FA, and 1.2 for RU. PRPE was stored at −80 °C until used for simulated GI model digestion studies.

### 2.2. In Vitro Gastrointestinal Digestion of Polyphenol-Rich Potato Extract 

PRPE was subjected to in vitro digestion by the GI model as described previously in detail [12]. Briefly, the model was composed of five double-jacketed vessels representing the stomach, the small intestine, the ascending colon, the transverse colon, and the descending colon. The model was fully computer controlled. The pH was automatically controlled by addition of 0.2 M HCl and 0.5 M NaOH solution upon a change in pH in the vessels to simulate the in vivo conditions in the different segments of the human gastrointestinal tract. The temperature was kept at 37 °C and anaerobic conditions were maintained in the fermentation vessels by flushing nitrogen gas for 20 min into the airspace every day. The experiment was started after a 2-week stabilization period in which fecal slurry, obtained from five healthy volunteers with no history of GI disease or antibiotic use in the previous six months, were inoculated into the last three vessels. After the 2-week stabilization period, 130 g PRPE was dissolved in the GI food mixture, which was composed of the following ingredients: arabinolactan, pectin, xylan, starch, glucose, yeast extracts, peptone, mucin, and cysteine powders—a composition previously developed by Molly et al. (1993) [27]. The GI food mixture was stored at 4 °C during the study. Samples were collected from all three colonic vessels before and 24 h after addition of PRPE to the GI digestion model. Each digestion was carried out in triplicate. Samples were centrifuged at 1000× *g* and stored at −80 °C for later analysis by liquid chromatography–mass spectrometry (LC-MS) and for the cell culture experiments. 

### 2.3. Sample Preparation for the Cell Culture Experiments

The samples that were collected from the last three colonic vessels of the gut model after 24 h digestion were pooled after each experiment and prepared according to the method described previously [28]. The samples were centrifuged at 36,000× *g*, 4 °C for 2 h. The supernatant was collected and the pH was neutralized to pH 7 before being filter sterilized with a 25 mm syringe filter (0.2 µm, MCE, Fisher Scientific, Ottawa, ON, Canada) and stored at −80 °C until used for the Caco-2/HepG2 cell experiments. Samples collected before the addition of PRPE were considered as the controls.

### 2.4. Cell lines and Culture Conditions 

Cells were obtained from the American Type Culture Collection (ATCC, Burlington, ON, Canada) and cultured according to the company’s procedures and Li et al. (2007) [23], as briefly explained below. The Caco-2 and HepG2 cells were cultured in Eagle’s minimum essential medium supplemented with 10% fetal bovine serum, 1% nonessential amino acids, 2 mM l-glutamine, and 0.1% penicillin-streptomycin mixture. Cells were incubated at 37 °C with 5% CO_2_ and 90% humidity and were monitored daily. The cells were subcultured at 80% confluence with a 0.25% trypsin-EDTA solution for 5–10 min and were cultured in a new flask or seeded onto an HTS Multiwell™ insert system with polyethylene terephthalate membrane (12 wells, 0.4 μM pore size, 1.12-cm^2^ area).

### 2.5. Caco-2/HepG2 Co-Culture System

Caco-2 cells were seeded onto inserts at a density of 60,000 cells/cm^2^ and were grown for 21 days under the same incubation conditions mentioned above. During this time, the medium was changed every other day. Before starting the treatments, the integrity of the monolayer was checked by measuring the transepithelial electrical resistance (TEER). A dose-response experiment was carried out in order to determine the optimal dose of the digesta with the minimum effect on the Caco-2 cells’ tight junctions as measured by TEER. Caco-2 monolayers having reached a TEER of 250 ohm/cm^2^ or greater were used for the transport experiment [29]. As the concentration of 10% (*v*/*v*) digesta in cell culture medium resulted in a minimal decrease in TEER (data not shown), this concentration was used to investigate the transport and metabolism of phenolic compounds by the Caco-2/HepG2 system. HepG2 cells were added to the basolateral side of the insert system at a concentration of 1 million cells/mL (1.5 mL). Medium (500 µL) containing digesta of PRPE was added to the apical side of the wells and incubated for up to 2 h. After 2 h, the donor plate was removed and the incubation was continued for 3 h with HepG2 cells in the receiver compartment. Samples were taken from the receiver well after 2 and 5 h. After incubation of the 10% digesta of PRPE with Caco-2/HepG2 cells, the supernatant of each compartment (apical and basolateral) was collected at different time points. All samples were centrifuged at 2000× *g* for 15 min and the supernatants were stored at −20 °C for further analysis by LC-MS. 

### 2.6. LC-MS Analysis for Identification of Phenolics Using Targeted Metabolite Analysis

After thawing, samples were vortexed and filtered using 25 mm syringe filters (0.45 µm, MCE, Fisher Scientific, Ottawa, ON, Canada) and transferred to HPLC vials for LC-MS analysis. Phenolic compounds were separated using a reverse phase column Gemini-NX (5 µm, 100 mm × 4.6 mm) (Phenomenex, Torrance, CA, USA) with a 4.6 mm × 2.0 mm guard column based on a method developed by Shakya and Navarre (2006) [30] and modified in our previous work [12]. Phenolic compounds and metabolites were eluted using a gradient of solvent A (10 mM formic acid, pH 3.5) and B (5 mM ammonium formate solution in 100% methanol), starting with 5% B, increasing to 30% B in 5 min, 70% B in 7 min, and 100% B at 9 min. This condition was maintained for 3 min. A solvent flow rate of 1.0 mL/min was used and 20 µL of sample was injected into the LC system. Accurate mass data were obtained using an Agilent 1200 series HPLC system equipped with an Agilent 6210 electrospray ionization, time-of-flight (ESI-TOF) mass spectrometer (Agilent, Santa Clara, CA, USA), with internal mass calibration. The analyses were performed in both positive and negative modes and the data was acquired over a mass range of *m*/*z* 100–1000. The source parameter settings were: temperature 350 °C, gas flow 12 L/min, nebulizer 50 psi capillary voltage (+/−) 4000 V, fragmentor 100 V, and skimmer voltage 60 V. Reference masses (internal calibration of high resolution spectra) were *m*/*z* 121.050873, 922.009798 for positive mode and *m*/*z* 119.03632, 966.000725 for negative mode. The data were acquired and processed using Agilent Mass Hunter software version B.04.00. Extracted ion chromatograms of accurate masses for deprotonated (MH−) or protonated (MH+) ions were used for confirmation of the presence of parent phenolic compounds as well as metabolites (within 10 ppm). The relative abundance of the compounds was calculated relative to the quantification of benzoic acid as the reference peak, as benzoic acid was present in all the cellular compartments at the same concentration at all time points.

## 3. Results and Discussion

### 3.1. Composition of the Digesta of PRPE Identified by Targeted Metabolite Profiling

The phenolic profiles from the pooled digesta from the three colonic vessels of the PRPE are shown in Table 1. The four parent phenolics (CGA, CA, FA, and RU), as well as 12 microbial-derived metabolites, were detected including dihydroferulic acid, dihydrocaffeic acid, 3-hydroxyphenylpropionic acid, coumaric acid, 3-hydroxyphenylacetic acid, phenylpropanoic acid, cinnamic acid, 3-hydroxybenzoic acid, and benzoic acid. This result is consistent with previous in vitro digestion studies showing that phenylpropionic, benzoic, phenylacetic, and cinnamic acids were the main metabolites generated after gut microbiota-mediated digestion of the above four parent phenolic compounds [12]. 

### 3.2. Transport and Metabolism of Phenolic Compounds by Caco-2/HepG2 Cells 

The profile of phenolic compounds present in the co-culture system incubated with 10% digesta of PRPE is shown in Table 2. A total of 10 microbial-derived metabolites were detected in the digesta of PRPE when this was diluted to a 10% concentration to maintain TEER above 250 ohms/cm^2^ for the transport studies. The presence of some microbial metabolites in control digesta incubated with the cell culture medium before PRPE addition (Table 3) may have arisen from the microbial fermentation of proteins and carbohydrates found in the GI nutrient medium [34] or could be explained by the lack of a polyphenol-restricted diet prior to the collection of the fecal samples. The presence of these metabolites has been reported previously in human fecal water [35]. 

FA was the only parent polyphenol detectable in the 10% digesta (Table 2). In agreement with previous Caco-2 cell transport studies, FA exhibited low permeability across the Caco-2 cell monolayer, which involves a concentration-dependent process involving a monocarboxylic acid transporter [29,36]. After the 2-h Caco-2 cell incubation, a high proportion of the initial FA concentration in the digesta was present in the apical side (90%) while only 11% was located in the basolateral side. Similarly, Koinishi and Shumizu (2003) reported that only a small percentage of FA (3.42%) was transported to the basolateral side of the Caco-2 cell monolayer [29]. 

Following the 2-h Caco-2 cell incubation, the apical side showed 78% and 67% of initial digesta concentrations of dihydroferulic acid and dihydrocaffeic acid, respectively. Those metabolites were the most predominant in the basolateral compartment showing 15% and 10% of the digesta concentrations for dihydroferulic acid and dihydrocaffeic acid, respectively. The presence of dihydroferulic acid could be partly attributable to Caco-2 cell-mediated biotransformation of FA as dihydroferulic acid has been indicated to be the main metabolite of Caco-2 metabolism of FA [37]. The observed concentration in the basolateral side for dihydrocaffeic acid was higher in comparison to a previous study that reported a permeability of 0.5% for dihydrocaffeic acid across Caco-2 cells involving a 1-h co-culture of Caco-2 and HT29-MTX cells [37]. The differences between studies might be attributable to the presence of mucus in the latter study, which can affect Caco-2 cell tight junctions and permeability [37]. Alternatively, or in addition, it is conceivable that absorption of dihydrocaffeic acid in the present work may have been enhanced by the concurrent presence of other microbial phenolic metabolites in the fecal digesta [38]. 

3-Hydroxyphenylpropionic acid was the predominant metabolite in the digesta of PRPE incubated with Caco-2 cells. The apical side showed that 69% of the digesta concentration was present after 2 h but only 4% of the initial concentration was noted in the basolateral side. The transport of 3-hydroxyphenylpropionic acid across the Caco-2 cell monolayer has been previously reported [39]. Likewise, 3-hydroxybenzoic acid and coumaric acid appeared to be poorly transported across Caco-2 cells. 3-Hydroxybenzoic acid and coumaric acid showed respective apical concentrations of 50% and 68% from values observed in the 10% digesta but basolateral concentrations were only 5% and 3% of the initial digesta values, respectively. The transport of *m*-coumaric and *p*-coumaric acids across Caco-2 cells via the monocarboxylic acid transporter has been previously reported with bidirectional transport of the apical to basolateral sides [39,40]. The relatively low concentration of coumaric acid in the basolateral compartment could be partly due to the basolateral to apical transport reported for coumaric acid in the absence of a proton gradient [40]. 

A major proportion (65–70%) of 3-hydroxyphenylacetic, phenylpropanoic, and cinnamic acids of their initial digesta concentrations was detectable in the apical side after the 2 h incubation but none of these three compounds were measurable in the basolateral component. Their absence in the basolateral side could be due to either low permeability across Caco-2 cells or further metabolism by Caco-2 cells. In concert with this observation, Konishi (2005) has reported that 99% of apically loaded hydroxyphenylacetic acid isomers remained in the apical side of Caco-2 cells, which was suggested to be due to their restricted transport via tight junctions [41]. 

Relative to the initial FA values in the basolateral compartment, the incubation with HepG2 cells alone for 3 h at the basolateral side led to a major 166% (1.6-fold) increase in FA concentration (Table 2). This increase could be accountable via hepatic-mediated dihydroferulic acid dehydrogenation as reported by Poquet et al. (2008) who showed the generation of FA from dihydroferulic acid after 30 min metabolism by rat liver slices [37]. Such metabolism can be partly responsible for circulating free FA in addition to a small percentage of glucuronide and sulfate FA conjugates [37]. The present work thus supports the contention that a significant proportion of plasma FA originates from in situ hepatic generation [42,43], particularly in view of its poor absorption seen in the current study and previous research [29]. 

After the 3 h incubation with HepG2 cells, 78% of the initial basolateral dihydroferulic acid concentration was observed. This value could be partly mediated by hepatic hydrogenase enzymes shown to produce dihydroferulic acid from FA [44]. Dihydroferulic acid appears mainly in plasma in an unconjugated form and hydroxycinnamate metabolism has been suggested to be responsible for plasma accumulation of dihydroferulic acid over time [11]. A remarkable three-fold increase in dihydrocaffeic acid concentrations in the basolateral compartment was seen following the HepG2 cell incubation. This increase could be partly mediated via hepatic dehydrogenation and *O*-demethylation reactions shown to generate dihydrocaffeic acid from FA and dihydroferulic acid [44]. Although not measured in the present study, both dihydrocaffeic and dihydroferulic acids could also undergo further intestinal and hepatic metabolism to generate glucuronidated and sulfated metabolites [45]. 

An increase of 233% (2.3-fold) in the basolateral concentrations of 3-hydroxyphenylpropionic acid was observed after the 3 h incubation with HepG2 cells, which supports hepatic metabolism as a major contributor to circulating concentrations of this metabolite. 3-Hydroxyphenylpropionic acid has been noted to be one of the most abundant microbial phenolic metabolites in plasma, urine, and fecal samples following polyphenol ingestion in human and animal feeding studies [10,42]. More than a two-fold increase in coumaric acid concentrations was seen in the basolateral compartment after a 3-h incubation with HepG2 cells, which could be partly due to the generation of coumaric acid from 3-hydroxyphenylpropionic acid by hepatic metabolism as previously suggested [10]. The 3-hydroxybenzoic acid concentration was unchanged in the basolateral compartment post-incubation with HepG2 cells, which suggests minimal hepatic degradation or biotransformation. The present results indicate that 3-hydroxybenzoic acid, one of the major products of gut microbiota metabolism, can be present following post-intestinal and hepatic metabolism. This finding coincides with the appearance of 3-hydroxybenzoic acid in the urine and plasma after consumption of polyphenol-rich diets [43].

## 4. Conclusions

In summary, the present results revealed that regardless of the poor Caco-2 cellular transport of FA and the microbial phenolic metabolites of dihydroferulic, dihydrocaffeic, 3-hydroxyphenylpropionic, 3-hydroxybenzoic, and coumaric acids, a notable increase in the basolateral concentrations of those compounds was observed following metabolism with HepG2 cells. The present data thus support the importance of post-prandial hepatic metabolism as a major source of circulating phenolic metabolites. It is noteworthy that in vivo feeding studies involving polyphenols have shown a gradual post-prandial appearance of plasma microbial phenolic metabolites at 0.5–4 h followed by a secondary increase in those metabolites at 4 h, which could thus be mediated by hepatic metabolism [46,47]. Future studies involving the combination of in vitro digestion models and Caco-2/HepG2 cell culture studies could also focus upon the production of sulfated, glucuronidated, or methylated metabolites of polyphenols that are predominant in the circulation [48]. Taken together, the results of the present study signify the importance of considering hepatic metabolism as a major contributor towards circulating phenolic metabolites following the intake of polyphenol-rich food products.

## Figures and Tables

**Table 1:** Polyphenols and their metabolites after human simulated intestinal digestion of polyphenol-rich potato extract (PRPE) ^1^.

Theoretical Mass (*m*/*z*) ^2^	Measured Mass	Mass Accuracy (ppm)	Retention Time (min)	Common Name	Systematic Name	PRPE
609.1461	609.1422	6.4	8.7	Rutin	Quercetin-3-*O*-rutinoside	+
353.0878	353.0863	4.3	7.5	Chlorogenic acid	(1*S*,3*R*,4*R*,5*R*)-3-{[(2*E*)-3-(3,4-Dihydroxyphenyl)prop-2-enoyl]oxy}-1,4,5-trihydroxycyclohexanecarboxylic acid	+
301.0354	301.0395	13.7	8.0	Quercetin	2-(3,4-Dihydroxyphenyl)-3,5,7-trihydroxy-4*H*-chromen-4-one	T
195.0663	195.0642	10.6	8.6	Dihydroferulic acid	3-(4-Hydroxy-3-methoxyphenyl)propionic acid	+
193.0506	193.0507	0.5	8.5	Ferulic acid	(E)-3-(4-Hydroxy-3-methoxy-phenyl)prop-2-enoic acid	+
181.0506	181.0509	1.6	7.0	Dihydrocaffeic acid	3-(3′,4′-Dihydroxyphenyl)propionic acid	+
179.0325	179.341	8.9	8.0	Caffeic acid	3,4-Dihydroxycinnamic acid	+
167.0350	167.0349	0.5	6.6	Vanillic acid	4-Hydroxy-3-methoxybenzoic acid	+
165.0557	165.054	10	8.4	3-Hydroxyphenylpropionic acid	3-(3-Hydroxyphenyl)propionic acid	+
163.0401	163.0409	4.9	8.4	Coumaric acid	The isomer is not specified from our data	+
153.0193	153.0192	0.6	7.2	Protocatechuic acid	3,4-Dihydroxybenzoic acid	+
151.0401	151.0398	1.98	7.7	3-Hydroxyphenylacetic acid	3-Hydroxyphenylacetic acid	+
149.0608	149.0599	6.03	9.4	Phenylpropanoic acid	Phenylpropanoic acid	+
147.0452	147.0453	0.6	8.5	Cinnamic acid	3-Phenylprop-2-enoic acid	+
137.0244	137.0241	2.1	7.2	3-Hydroxybenzoic acid	3-Hydroxybenzoic acid	+
121.0295	121.0297	1.6	9.0	Benzoic acid	Benzoic acid	+

^1^ Determined by LC-MS analysis; ^2^ Identification based on previous literature data [18,31,32,33]; (+) present, (T) trace amount.

**Table 2:** Polyphenols and their metabolites in PRPE digesta (*t* = 0 h) and amount expressed as a percentage of initial digesta values in the apical (*t* = 2 h) and basolateral (*t* = 2 h) chambers in the Caco-2 cell transport bioassay and basolateral (*t* = 5 h) chamber in the HepG2 cell metabolism bioassay ^a^.

Theoretical Mass (*m*/*z*)	Measured Mass	Mass Accuracy (ppm)	Retention Time (min)	Common Name	PRPE			
					Digesta ^b^ (0 h)	Percentage of Digesta Value ^c^		Percentage of Basolateral Value ^d^
						A (2 h)	B (2 h)	B (5 h)
195.0663	195.0642	10.6	8.6	Dihydroferulic acid	1.22	78	10	78
193.0506	193.0507	0.5	8.5	Ferulic acid	1.07	90	11	166
181.0506	181.0509	1.6	7.0	Dihydrocaffeic acid	3.00	67	15	338
165.0557	165.054	10	8.4	3-Hydroxyphenylpropionic acid	15.11	69	4	233
163.0401	163.0409	4.9	8.4	Coumaric acid	2.44	68	3	212
151.0401	151.0398	1.98	7.7	3-Hydroxyphenylacetic acid	1.02	70	-	-
149.0608	149.0599	6.03	9.4	Phenylpropanoic acid	0.32	65	-	-
147.0452	147.0453	0.6	8.5	Cinnamic acid	0.31	67	-	-
137.0244	137.0241	2.1	7.2	3-Hydroxybenzoic acid	5.12	50	5	100
121.0295	121.0297	1.6	9.0	Benzoic acid	+	+	+	+

^a^ Identification based on previous literature data [18,31,32,33]; ^b^ Quantities of polyphenols and their metabolites in 10% (*v*/*v*) potato extract digesta are calculated relative to the concentration of benzoic acid. ^c^ Amount expressed as a percentage of initial digesta values in the apical chamber (A) and basolateral chamber (B) after 2 h in the Caco-2 cell transport bioassay. ^d^ Amount expressed as a percentage of initial digesta values in the basolateral chamber (B) after 3 h in the HepG2 metabolism bioassay. (+) present, (-) absent.

**Table 3:** Polyphenols and their metabolites in control digesta (*t* = 0 h) and amount expressed as a percentage of initial control values in the apical (*t* = 2 h) and basolateral (*t* = 2 h) chambers in the Caco-2 cell transport bioassay and basolateral (*t* = 5 h) chamber in the HepG2 cell metabolism bioassay ^a^.

Theoretical Mass (*m*/*z*)	Measured Mass	Mass Accuracy (ppm)	Retention Time (min)	Common Name	Control			
					Control ^b^ (0 h)	Percentage of Control Value at 0 h ^c^		Percentage of Basolateral Value ^d^
						A (2 h)	B (2 h)	B (5 h)
181.0506	181.0509	1.6	7.0	Dihydrocaffeic acid	1.1	26	26	123
165.0557	165.054	10	8.4	3-Hydroxyphenylpropionic acid	0.53	90	-	-
151.0401	151.0398	1.98	7.7	3-Hydroxyphenylacetic acid	0.42	100	-	-
149.0608	149.0599	6.03	9.4	Phenylpropanoic acid	0.28	100	-	-
121.0295	121.0297	1.6	9.0	Benzoic acid	+	+	+	+

^a^ Identification based on previous literature data [18,31,32,33]; ^b^ Quantities of phenolic metabolites in controls are calculated relative to the concentration of benzoic acid. ^c^ Amount expressed as a percentage of initial values of control samples in the apical chamber (A) and basolateral chamber (B) after 2 h in the Caco-2 cell transport bioassay. ^d^ Amount expressed as a percentage of initial values of controls in the basolateral chamber (B) after 3 h in the HepG2 metabolism bioassay. (+) present, (-) absent.
